# Decomposing the effects of digitalization on workers’ job satisfaction

**DOI:** 10.1007/s12232-022-00392-6

**Published:** 2022-04-17

**Authors:** Thomas Bolli, Filippo Pusterla

**Affiliations:** 1 Chair of Education System, ETH Zurich, Leonhardstrasse 21, 8092 Zurich, Switzerland; 2 Swiss Federal University for Vocational Education and Training, Kirchlindachstrasse 79, 3052 Zollikofen, Switzerland

**Keywords:** Digitalization, Job satisfaction, Professional education and training, J28, O33

## Abstract

This paper provides novel results on the relative importance of multiple channels through which digitalization affects job satisfaction. Using part-time students and graduates of professional education and training colleges in Switzerland as a case study, we investigate the relative strength of ten different channels. We find that the association between digitalization and job satisfaction is positive on average. This relationship is mainly due to the increase in productivity and more interesting work. Heterogeneity analyses on subsets of workers suggest that the effect through increasing productivity is more beneficial for women, for older workers, for workers without an executive position, and for workers who did not study in technology-related fields. The effect through the interestingness of work is larger for males and for older workers. Our results further suggest that among the channels that decrease job satisfaction, increase of time pressure and worsening of work-life balance are much more important than the threat of losing one’s job. Both channels are more relevant for men, for older workers, and for workers whose field of study is technology-related.

## Introduction

Digitalization is the rapidly growing sociotechnical phenomenon of adopting information and communication technologies (ICT) (Legner et al. 2017). Most of the economic literature analyzing the labor market effects of digitalization focuses on the number of jobs that new technologies replace (e.g., Degryse 2016; Frey and Osborne 2017; Arntz et al. 2020). Yet relatively little attention has been paid to the effects of ICT adoption on jobs not replaced by digitalization, with only limited evidence on the mechanisms through which digitalization affects workers’ job satisfaction.

However, firms’ ability to assess the way in which digitalization affects job satisfaction is crucial, because understanding through which channels digitalization affects workers’ job satisfaction might help them better evaluate the introduction of new technologies. Likewise, workers’ knowing how digitalization will affect their job satisfaction might help them to assess the consequences of increasingly diffuse work practices (e.g., home offices).

Theoretically, digitalization can affect workers’ job satisfaction either positively (e.g., by decreasing the percentage of repetitive tasks and increasing that of interesting ones) or negatively (e.g., by increasing the level of stress or decreasing work-life balance). A growing body of literature at the intersection of economics and psychology suggests an overall positive effect of digitalization on workers’ job satisfaction and well-being (e.g., McMurtrey et al. 2002; Salanova et al. 2004; Golden and Veiga 2005; Day et al. 2010; Limbu et al. 2014). However, no study looking at how digitalization might affect job satisfaction has yet examined more than one channel through which that effect might operate. For example, Moqbel et al. (2013) highlight the role of social networks in increasing workers’ job satisfaction, while Martin and Omrani (2015) show that information technology use positively affects job satisfaction due to an increase in labor productivity. Thus far, no paper systematically identifies and assesses the multiple channels through which digitalization affects job satisfaction.

This paper investigates ten channels through which digitalization may affect job satisfaction simultaneously. In all channels, digitalization affects job satisfaction by first changing some characteristic of the job itself, and then that change impacts the worker’s job satisfaction. Therefore, all of the channels through which digitalization might affect job satisfaction are changes in job characteristics caused by digitalization. Based on the theoretical framework of Castellacci and Tveito (2018), we identify ten channels through which digitalization affects job satisfaction.

Specifically, we expect that digitalization decreases job satisfaction by increasing time pressure at work, by increasing the fear of losing one’s job, by deteriorating work-life balance, and by smoothing the transition between working hours and leisure time. Conversely, we expect that digitalization increases job satisfaction by making work more interesting, by reducing the proportion of repetitive tasks, by increasing productivity, and by increasing autonomy at work. Furthermore, we expect that digitalization also increases job satisfaction by making forms of working more flexible and by simplifying interactions with colleagues and superiors.

We empirically evaluate the relative importance of the ten channels by using a survey conducted among part-time students and graduates of professional education and training (PET) colleges in Switzerland in 2019. Beyond general information on workers, our survey contains specific questions on digital transformation in the workplace, including asking respondents to evaluate statements about the effects of digitalization on different job characteristics and to self-assess the effect of digitalization on their job satisfaction. Respondents relate digitalization to the use of ICT, as well as the use of data and software in business processes. Having information on both the total effect of digitalization on job satisfaction and the effect of digitalization on single job characteristics allows us to assess the relative importance of the channels through which digitalization affects job satisfaction.

Our results suggest that digitalization is positively associated with worker’s job satisfaction of PET graduates through the following channels: increase in work productivity, more interesting work, simpler interactions with coworkers and supervisors, grater workers’ autonomy, and flexible forms of work are positively associated with workers’ job satisfaction. Furthermore, our results suggest that an increase in time pressure derived by digitalization has only a moderate negative effect on job satisfaction. However, we find that the worsening work-life balance derived by digitalization but not by smoothing the transition between working hours and leisure time negatively affects job satisfaction. Finally, our estimates provide no evidence the reduction of repetitive tasks caused by digitalization has an effect on workers’ job satisfaction. Although the widespread notions that the fear of losing one’s job to digitalization negatively affects job satisfaction is confirmed, it remains small in magnitude in our sample.

Furthermore, heterogeneity analyses on subset of workers suggest that the worsening of the work-life balance is more relevant for men, for workers aged more than 35 years (roughly the average age in our sample), for workers with an executive position, and for workers whose field of study is technology-related. For the interestingness of work, we find a larger effect for males and for workers older than 35. In contrast, the effect that digitalization has on job satisfaction through an increase in autonomy is lower for women, for young workers, and for workers who did not study in technology-related fields. In terms of productivity, we find that digitalization is more beneficial for women, for older workers, for workers without an executive position, and for workers who did not study in technology-related fields. Finally, the positive effect that digitalization has on job satisfaction by simplifying interactions with colleagues and superiors is larger for non-executive workers than for executives.

This paper empirically contributes to the current debate on the impact of digitalization on job satisfaction. Concretely, we provide first evidence on the relative importance that these channels have in explaining the effect of digitalization on workers’ job satisfaction.

The rest of the paper is organized as follows. Section 2 reviews the literature and describes the ten channels. Section 3 explains the estimation strategy, and Sect. 4 describes the data set. Section 5 presents the results and discusses the heterogeneity across workers. Section 6 concludes and discusses implications for future research.

## Literature review

A growing body of literature at the intersection of economics and psychology suggests a positive relationship between digitalization and workers’ job satisfaction (e.g., McMurtrey et al. 2002; Salanova et al. 2004; Golden and Veiga 2005; Day et al. 2010; Limbu et al. 2014; Martin and Omrani 2015). However, no study in this literature has yet analyzed the channels through which digitalization affects job satisfaction in a comprehensive framework. To fill this gap, this paper decomposes the effects of digitalization on workers’ job satisfaction into different channels and assesses their importance relative to one another.

To identify the channels through which digitalization affects job satisfaction, we build on Castellacci & Tveito’s (2018) theoretical model, which groups these channels into four distinct dimensions. First, while digitalization increases efficiency and frees up time, it can make some occupations obsolete. Thus, digitalization has an effect on job satisfaction through the “change in time use” dimension. Second, digitalization can create new activities that provide both security and personal control, in turn leading to a positive effect on job satisfaction and well-being. Digitalization has thus an effect on job satisfaction through the “new activities” dimension.

Third, digitalization enables individuals to obtain, access, process, and archive information much more systematically and rapidly than previously possible. Easier information access improves quality of work and eventually workers’ job satisfaction. Digitalization thus has an effect on job satisfaction through the “access of information” dimension. Fourth, while digitalization increases the opportunities for communication and eventually fosters social capital and knowledge sharing, it also distracts workers and reduces their efficiency. Digitalization thus has an effect on job satisfaction through the “communication tools” dimension.

Castellacci and Tveito (2018) provide a description of the four dimensions in their theoretical framework. To understand the mechanisms through which digitalization affect workers’ job satisfaction, we disentangle these descriptions into ten specific channels. Each of these describes one job characteristics affected by digitalization, and the changes in job characteristics drive changes in workers’ job satisfaction. Specifically, we consider the following ten job characteristics affected by digitalization as channels through which digitalization affects job satisfaction: Time pressure, fear of losing one’s job, work-life balance, smoothness of transition between work and private life, interestingness of tasks, productivity, autonomy, working time flexibility, and the simplicity of interaction with colleagues and superiors. For simplicity, hereafter we refer to the effect that digitalization has on job satisfaction through a job characteristic as “channels.” A channel describes thus digitalization’s effect on job satisfaction via a change in a specific job characteristic.

**Fig. 1:**
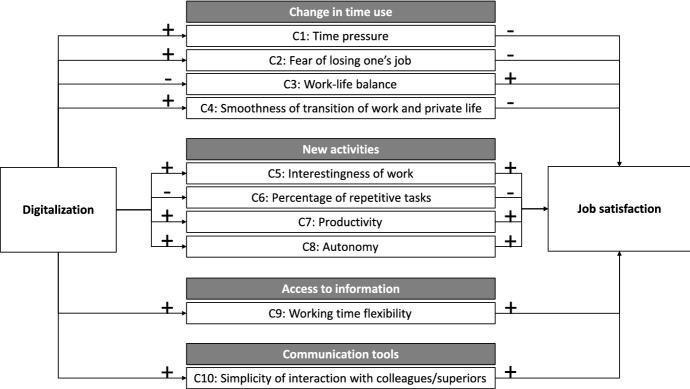
Summary of the channels. *Notes*: This figure summarizes the channels through which digitalization affects job satisfaction investigated by this paper. For each channel, the figure displays the effect of digitalization on the job characteristic and of the job characteristic on job satisfaction. “+” suggests a positive relation, while “−” a negative one

Figure 1 previews the channels that we investigate in this paper. The ten job characteristics are grouped according to the four dimensions developed by Castellacci and Tveito (2018). The left side shows the effect of digitalization on the ten job characteristics (e.g., the effect of digitalization on time pressure at work). The right side displays the effect of the ten job characteristics on job satisfaction (e.g., the effect of time pressure at work on job satisfaction). The combination of these effects yields the effect of digitalization on job satisfaction for each job characteristic and, therefore, each channel.

By describing the channels, we refer on the entire sample and refrain from refining them according to workers’ individual characteristics. Nevertheless, in Sect. 5.2 we present and discuss the heterogeneity of our results according to gender, age, management position, and field of study.

### Change in time use

The dimension change in time use includes four channels (in our model, 1–4): time pressure, fear of job loss, work-life balance, and transition smoothness between work and private life. The first channel, time pressure, captures the possibility that digital technologies at work can expose employees to working under pressure, having frequent tight deadlines resulting from electronic workflows, and lacking sufficient time for carrying out daily tasks (Agypt and Rubin 2012). Additionally, recent studies also evidence a large effect of email interruptions (Mark et al. 2016; Stich et al. 2019) and smartphone notification (Fitz et al. 2019) on the perceived work stress. Similarly, Mullan and Wajcman (2019) found a significant—though small—impact of mobile devices on longer working hours, and evidence that it was significantly associated with time pressure. These conditions create “technostress,” which Tarafdar et al. (2010) define as the psychological effects stemming from the inability to cope with computer or software use at work. A large literature shows that technostress negatively affects job satisfaction (e.g., Tarafdar et al. 2007; Ragu-Nathan et al. 2008; Ayyagari et al. 2011). We therefore summarize this channel as follows:

C1: The increase in time pressure at work generated by digitalization is negatively associated with job satisfaction.

The second channel, fear of job loss, captures the likelihood that digitalization will make certain jobs obsolete (Rotman 2013; Autor 2014, 2015). Research shows that the perception of job insecurity is an important factor in stress (Hartley et al. 1990), which is negatively related to job satisfaction (Reisel et al. 2010). Brougham and Haar (2018) show that employees awareness of technological advancements such as smart technology, artificial intelligence, robotics, or the diffusion of algorithms is negatively related to organizational commitment and job satisfaction. We therefore summarize this channel as follows:

C2: The increase in the fear of losing one’s job derived by digitalization is negatively associated with job satisfaction.

As the fear of losing one’s job is caused by changes in tasks that are performed by humans, we follow Castellacci and Tveito (2018) and classify this channel under the change in time use dimension.

The third channel, work-life balance, captures the deterioration of work-life balance created by digitalization (Nam 2014). The literature suggests that a worse work-life balance negatively affects subjective job satisfaction (Gallie and Russell 2009; Scandura and Lankau 1997). For instance, Derks et al. (2015) show that smartphone use increases work–home interference, as employees think their superiors expect them to always be available. Additionally, Dettmers et al. (2016) evidence that daily extended work availability has a negative effect on workers psychological and physiological well-being, as employees which are required to remain available outside working hours are constrained in their recovery from work. We therefore summarize this channel as follows:

C3: The deterioration of the work-life balance induced by digitalization is negatively associated with job satisfaction.

The fourth channel, transition smoothness between work and private life, captures digitalization’s allowing a smoother transition between working hours and leisure time (Boswell and Olson-Buchanan 2007). A smoother transition between the two can have negative consequences for job satisfaction, for example, by exacerbating the work-family conflict (Boswell and Olson-Buchanan 2007). We therefore summarize this channel as follows:

C4: The smoothing of the transition between working hours and leisure allowed by digitalization is negatively associated with job satisfaction.

### New activities

The dimension new activities includes four channels (in our model, 5-8): interestingness of tasks, percentage of repetitive tasks, productivity, and autonomy. The first of these channels (i.e., the fifth channel), interestingness of tasks, captures the way in which digitalization leads to the creation and development of new working activities and tasks (Carlsson 2004). These new activities often require specific skills, provide physical security, and increase personal control, all factors that are positive for job satisfaction (Warr 2003; Castellacci and Viñas-Bardolet 2019). We therefore summarize this channel as follows:

C5: The increase in work interestingness derived from digitalization is positively associated with job satisfaction.

The sixth channel, percentage of repetitive tasks, captures the effect of digitalization in reducing the proportion of repetitive tasks and physically straining labor (Acemoglu and Autor 2011). Such a reduction allows workers to allocate more time to more rewarding activities (Askenazy and Caroli 2010), an outcome that, in turn, has a positive effect on workers’ job satisfaction (Melamed et al. 1995; Kristensen and Johansson 2008). Nevertheless, the introduction of new tasks might also increase the level of job stress, in turn negatively influencing job satisfaction (Konradt et al. 2003; Morris and Venkatesh 2010). However, Castellacci and Viñas-Bardolet (2019) suggest that the effect of a reduction in repetitive tasks on job satisfaction is particularly positive for white-collar workers. As the survey sample in this paper consists of tertiary-educated workers, we favor the argument of more rewarding activities. We thus formulate the channel as follows:

C6: The reduction in the proportion of repetitive tasks induced by digitalization is positively associated with job satisfaction.

The seventh channel, productivity, shows that digitalization allows more productive activities (Brynjolfsson and McAfee 2011). Activities that are more productive imply higher wages (all else being equal), which in turn lead to higher job satisfaction (D’Addio et al. 2007; Castellacci and Viñas-Bardolet 2019). As Barrero et al. (2021) highlight, such a mechanism has been recently observed during the COVID-19 pandemic, when new technologies allowed a re-optimization of working arrangements, which in turn ended up in increased workers’ productivity. We therefore summarize this channel as follows:

C7: The increase in productivity generated by digitalization is positively associated with job satisfaction.

The eighth channel, autonomy, captures the effect of digitalization on employees’ autonomy at work. Bloom et al. (2014) suggest that information technologies and communication technologies have different effects with regard to autonomy. On the one side, information technologies make accessing information less expensive, thereby giving workers more autonomy and a wider span of control. On the other side, communication technologies reduce communication costs and therefore lead to more centralized management. Communication technologies thus act as a centralizing force that reduces workers autonomy.

However, Mazmanian (2013) provides evidence that digital devices do not limit workers’ discretion, freedom, or authority but instead enhance their autonomy. Similarly, Martin (2017) links ICT use with workers’ autonomous motivations and shows that ICT, by facilitating access to information such as through workflow, Internet, and e-mail, most contributed to the development of a motivational environment. Furthermore, the literature shows that workers with a higher degree of autonomy are typically more satisfied (Golden and Veiga 2005; Lopes et al. 2014).

We therefore summarize this channel as follows:

C8: The increase in the autonomy at work derived by digitalization is positively associated with job satisfaction.

### Access to information

The dimension access to information has only one channel (in our model, 9). This ninth channel, working time flexibility, captures the way digitalization improves employees’ access to information, with an increasing number of tasks no longer requiring a specific workstation (Popma 2013). Workplace-independent access to information enables more flexible working time (Duxbury et al. 2007), and Raziq and Maulabakhsh (2015) show that flexible working hours increase job satisfaction. Similarly, Kelliher and Anderson (2010) find that flexible workers report higher levels of job satisfaction than their non-flexible counterparts. We therefore summarize this channel as follows:

C9: More flexible forms of work allowed by digitalization are positively associated with job satisfaction.

Following Castellacci and Tveito (2018), we assign this channel to the access to information dimension as digitalization ease the access to information when not present at the workplace enabling thus working time to become more flexible.

### Communication tools

The dimension communication tools have only one channel (in our model, 10). This tenth channel, simplicity of interaction with colleagues and superiors, captures digitalization’s simplifying the interactions between individuals. For example, Koku et al. (2001) highlight the positive effect of the Internet in facilitating and maintaining off-line relationships. Zhao (2006) finds that individuals using the Internet for interpersonal contact usually have more social ties than those who do not. Furthermore, digital technologies also simplify workplace interaction. Moqbel et al. (2013) focus on the role of social networking sites (SNS), which are web-based services that allow workers to build social networks or relationships with other people. They find that the use of SNS at work increases organizational commitment and job satisfaction.

In a sample covering 13 countries, Amichai-Hamburger and Hayat (2011) investigate the influence of Internet use on social interactions, finding that Internet usage is positively correlated with the socially related interactions of people in the same profession. In turn, simplified interactions with colleagues and superiors increase workers’ job satisfaction (Pincus 1986; Warr 2003). Additionally, Intranet use at work has been found to positively affect the sharing of internal knowledge within a firm (Hendriks 1999), and knowledge sharing improves social capital (Huysman and Wulf 2006) and increases work quality (Haas and Hansen 2007), in turn increasing job satisfaction (Requena 2003).

Nevertheless, some recent studies also show that the use of communication tools at work (e.g., Facebook) can have negative effects on productivity, in turn negatively affecting workers’ morale and job satisfaction (Brooks 2015). However, despite these new contradictory results, we favor the argument of enhanced communication because more largely documented by the literature. We therefore summarize this channel as follows:

C10: More simply interaction with colleagues and superiors eased by digitalization are positively associated with job satisfaction.

## Empirical strategy

This section presents the empirical strategy we use to assess the relative importance of the channels through which digitalization affects job satisfaction. To do so, we start by presenting a structural model and discussing the challenges that such an approach could pose. We then apply a reduced-form model, which allows us to assess the relative importance of the channels and poses fewer challenges to both measuring digitalization intensity and assessing job satisfaction.

### Structural model

Identifying the influence of each channel in a structural model requires estimating multiple equations. First, we need to estimate the effect of digitalization on ten job characteristics for worker *i* as represented by the following system of equations:

1 $$\begin{aligned} Job~Characteristic_{i}^{c} =\phi _{c}+\theta _{c}~Digitalization_{i}+\vartheta _{c}~X_{i}+\tau _{ci} \end{aligned}$$

where $Digitalization_{i}$ stands for the digitalization of worker *i*’s job, $X_{i}$ is a vector of other variables that affect job satisfaction of worker *i*, and $\tau$ is the error term.

Second, the structural model contains an estimation of the relationship between the ten job characteristics *c* and the job satisfaction of worker *i*:

2 $$\begin{aligned} Job~Satisfaction_{i} = \alpha + \sum _{c=1}^{10}\beta _{c}\,\,Job~Characteristic_{i}^{c}+\eta ~X_{i}+\vartheta _{i} \end{aligned}$$

where $\beta _{c}$ reflects the impact of job characteristic *c* on job satisfaction. The vector *X* is defined as above, while $\vartheta$ is the error term.

However, estimating this structural model faces a number of challenges in terms of measuring the variables in Eqs. 1 and 2. An empirical challenge involves the difficulty in measuring digitalization. The literature often measures digitalization by counting the number of computer or digital devices (Caselli and Coleman 2001). Nevertheless, the stock of computers measures digitalization imperfectly, because it measures only the availability of computers, not their effective use by workers. Therefore, we apply a reduced-form model, which allows us to identify the relative importance of each channel, by analyzing the relationship between digitalization and job satisfaction directly rather than measuring digitalization and job satisfaction separately.

### Reduced-form model

Inserting Eq. 1 into Eq. 2 and taking the first derivative with respect to digitalization yields the following reduced form:

3 $$\begin{aligned} \omega _{i} = \frac{\partial {Job~Satisfaction_{i}}}{\partial Digitalization_{i}} = \sum _{c=1}^{10}\beta _{c} \theta _{ci} +\epsilon _{i} \end{aligned}$$

where $$\omega _{i}$$ is the partial derivative of job satisfaction with respect to digitalization. $$\theta _{c}$$ denotes the effect of digitalization on job characteristic *c*. $$\beta _{c}$$ reflects the impact of job characteristic *c* on job satisfaction. $$\beta _{c}$$$$\theta _{c}$$ represents the impact of digitalization on job satisfaction through job characteristic *c*.

We operationalize $\omega _{i}$ by asking respondents how strongly digitalization affects his or her job satisfaction, measured on a five-point Likert scale. This reduced-form model has the advantage that it does not require measuring digitalization and job satisfaction. However, the reduced-form model has the disadvantage that the definition of digitalization depends on the interpretation of the respondents. An open-ended question in which we asked what respondents understand by the term digitization reveal that the majority of them relate digitalization to the use of information and communication technology, as well as the use of data and software in business processes.

Similarly, we operationalize $\theta _{c}$ by asking respondents to assess the impact of digitalization on the corresponding job characteristic *c*. We thus estimate via OLS the following equation:

4 $$\begin{aligned} \widetilde{\omega _{i}} = \sum _{c=1}^{10}\beta _{c} \widetilde{\theta _{ci}} +\gamma \widetilde{X_{i}} +\epsilon _{i} \end{aligned}$$

where the superscript $\widetilde{~~~}$ describes parameters that have been self-assessed by respondents. This equation also accounts for other workers’ characteristics that might affect job satisfaction but are unrelated to digitalization. Specifically, $\widetilde{X_{i}}$ is a vector of control variables, a vector with the following worker characteristics: *age*, $age^{2}$, *gender*, a dummy for an executive position, 8 dummies for the field of study, and 13 dummies for the industry. Finally, the identification of $\beta_{c}$ assumes that job characteristics *c* are orthogonal to any other potential characteristics through which digitalization affects job satisfaction. To account for this potential source of omitted variable bias, $\widetilde{X_{i}}$ further includes a variable that captures how strongly respondents assess the impact of digitalization on their job in the previous year, measured on a five-point Likert scale. For example, a respondent reporting that digitalization strongly affected her job satisfaction may overestimate the overall impact of digitalization on her workplace and occupation. Controlling for this could also mitigate some possible endogeneity due to respondents’ subjective perceptions and evaluations. Lastly, $\epsilon$ is the error term that is estimated robust.

Estimating Eq. 4 via OLS yields estimates for the impact of job characteristic *c* on job satisfaction. To analyze the effect of digitalization on job satisfaction, we multiply for each worker characteristic *c* the estimated $\widehat{\beta _{c}}$ with the corresponding $\widetilde{\theta _{c}}$. While the calculation of $\widehat{\beta _{c}}$ $\widetilde{\theta _{c}}$ is straightforward, the interpretation of its magnitude is far from trivial. Indeed, this measure combines the effect of job characteristic c on job satisfaction ($\widehat{\beta _{c}}$) and the extent to which workers agree with their survey assessment of the impact of digitalization on this job characteristic ($\widetilde{\theta _{c}}$). Therefore, to simplify the interpretation, we decompose the overall goodness of fit $R^{2}$ into the explanatory power of individual regressors. The decomposition of $R^{2}$ translates into the importance of the different regressors by giving a measure that is more easily interpreted.

One convenient measure for decomposing the overall goodness of fit is the Shapley value (Shapley 1953), which computes the contribution of a single variable to the goodness of fit of a statistical model. Assume, for example, a full regression model with *k* explanatory variables ($x_{1}$, $x_{2}$, ..., $x_{k}$). According to Huettner et al. (2012), to calculate the contribution of each variable, we need to estimate all possible submodels derived by the permutation of the regressors. Mathematically, to calculate the contribution of a given regressor *j* we need to estimate the same number of submodels as the number of permutations (*K*!) of *k* regressors:

5 $$\begin{aligned} R^{2}_{j} = \frac{1}{K!} R^{2}(f(x_{j}^{\mu },x_{j}))- R^{2}(f(x_{j}^{\mu })) \end{aligned}$$

where $\mu$ maps all *K*! variable permutations. By subtracting the $R^{2}$ of the model not including $x_{j}$ from the $R^{2}$ of the model including $x_{j}$ and all regressors preceding $x_{j}$ in that particular order ($x_{j}^{\mu }$), we obtain the Shapley value, which measures *j*’s average marginal contribution to $R^{2}$ across all possible permutations.

In other words, we iteratively remove all regressor variables from the full model and estimate the goodness of fit of every (sub-)model. A variable’s marginal contribution is given by the difference in the goodness of fit associated with the elimination of a regressor. By giving equal probability in the order in which we remove the regressors, the Shapley value of a variable is given by the average marginal contribution over all possible orderings.

## Data and description of variables

The data stems from the ODEC Salary Survey conducted as an online survey among students (most of them in part-time education) and graduates of Swiss professional education and training (PET) colleges in 2019. This formal vocational tertiary education at level 6 of the ISCED-2011 classification takes from two to four years, depending on the PET college and on whether the education is full-time or part-time. About two third of the curricula are designed as part-time education, but also full-time curricula can be completed in part-time mode (BSS 2021). While (part-time) students account for about 10% of the sample and have a response rate of about 20%, graduates account for the remaining 90%, with a response rate of about 11%.^1^ Table 1 shows the summary statistics of variables used in the estimation. The dependent variable measures the in​uence of digitalization on job satisfaction on a five-point Likert scale (1 = “less satisfied”; 3 = “no change”; 5 = “more satisfied”). The mean of 3.47 suggests that digitalization on average increases job satisfaction of workers with a PET college diploma. This persistent positive effect of digitalization on workers’ job satisfaction—in line with the findings in the literature (e.g., McMurtrey et al. 2002; Salanova et al. 2004; Golden and Veiga 2005; Day et al. 2010; Limbu et al. 2014; Martin and Omrani 2015)—needs cautious interpretation, because it is specific to the subsample in this paper.

Breakdowns of the dependent variable by gender, age, management position, and field of study yield values above 3, suggesting an overall positive effect of digitalization on job satisfaction. Nevertheless, some differences are noteworthy. Men report a larger positive effect of digitalization on job satisfaction than women. Additionally, workers younger than age 35 also report higher levels of job satisfaction than do older workers. We also observe small differences between workers in executive positions, who report a slightly larger positive effect than non-executive workers. Finally, across fields of study, we find that workers in the fields of social work and adult education report almost no change in digitalization-induced job satisfaction, whereas workers from the fields of arts and business administration report a relatively large positive effect.

**Table 1:** Variables description

	*N*	Mean	SD	Min	Max
*Dependent variable*					
Dig. affects my job satisfaction	3089	3.47	0.91	1	5
*Main explanatory variables*					
C1: Dig. increases the time pressure at work	3089	3.23	1.18	1	5
C2: Dig. puts my job at risk	3089	1.94	1.06	1	5
C3: Dig. worsens the work-life balance	3089	2.72	1.18	1	5
C4: Dig. leads to a smooth transition between working hours and leisure time	3089	3.01	1.2	1	5
C5: Dig. makes my work more interesting	3089	3.4	1.09	1	5
C6: Dig. reduces the proportion of repetitive tasks	3089	3.24	1.16	1	5
C7: Dig. increases my productivity	3089	3.65	1.04	1	5
C8: Dig. increases my autonomy at work	3089	3.15	1.06	1	5
C9: Dig. enables more flexible forms of working time	3089	3.25	1.35	1	5
C10: Dig. simplifies interactions with colleagues and superiors	3089	3.41	1.11	1	5
*Control variables*					
How strongly does Dig. affect the work over the last year?	3089	3.44	1.14	1	5
Age	3089	35.7	9.65	20	72
Women	3089	0.19		0	1
Executive (dummy for being firm’s board director or member of management)	3099	0.29		0	1
*Field of study*					
Agronomy	3089	0.01		0	1
Catering	3089	0.05		0	1
Health	3089	0.06		0	1
Arts	3089	0.01		0	1
Social work and adult education	3089	0.04		0	1
Technology	3089	0.66		0	1
Business administration	3089	0.17		0	1
*Industry*					
Manufacturing	3089	0.33		0	1
Construction	3089	0.12		0	1
Wholesale and retail trade; repair of motor vehicles and motorcycles	3089	0.03		0	1
Transportation and storage	3089	0.03		0	1
Accommodation and food service activities	3089	0.04		0	1
Information and communication	3089	0.08		0	1
Financial and insurance activities	3089	0.05		0	1
Professional, scientific and technical activities	3089	0.06		0	1
Administrative and support service activities	3089	0.08		0	1
Public administration and defense; compulsory social security	3089	0.05		0	1
Education	3089	0.04		0	1
Human health and social work activities	3089	0.10		0	1
Other service activities	3089	0.00		0	1

We exemplify digitalization in the survey as, e.g., automatization and monitoring production processes, data analysis, and customer relationship management. Furthermore, we can analyze an open-ended question about how digitalization has affected their job. The results show that respondents understand digitalization as aspects related to information and communication technology. Typical responses refer to storage and analysis of data, information, and documents. Relatedly, respondents frequently mention planning, monitoring and communication processes, with a particular emphasis on the automatization and interlinkage of administrative processes. The most mentioned technologies are ERP, CAD, and Cloud. In contrast, robots and AI are rarely mentioned.

The main explanatory variables capture, on a five-point Likert scale, to what extent respondents agree with statements about the impact of digitalization on the ten job channels through which we expect that digitalization affects job satisfaction (1 = “ I don’t agree at all”; 5 = “ I fully agree”). The results suggest that the strongest effect of digitalization lies in increasing productivity (3.65), followed by simplifying interactions with colleagues and superiors (3.41) and making work more interesting (3.4). Moreover, we find an average effect in terms of an increase in more flexible forms of working time (3.25), a reduction in the proportion of repetitive tasks (3.24), an increase in time pressure (3.23), an increase in autonomy (3.15), and a smooth transition between working hours and leisure time (3.01). The least strong effects appear in terms of worsening work-life balance (2.72) and fear of losing one’s job (1.94).

If digitalization has only a moderate effect on a given job characteristic, we hardly identify the overall effect of digitalization on job satisfaction through this job characteristic, and thus independently on the effect that this job characteristic has on job satisfaction. Therefore, these results cast doubt on both channels C3 and C5. However, these two low values do not necessarily mean that work-life balance and the fear of losing one’s job have no effect on job satisfaction. Instead, it means that digitalization does not affect them.

The control variables in the bottom part of Table 1 show that most respondents are male and between ages 20 and 72. The average age of respondents is about 36 years, meaning that our sample is relatively young. About 30% hold executive positions, either as a member of a firm’s board of directors or as part of management. The summary statistics show that about two-thirds of respondents chose technology-related field of study. About one-sixth are in business administration, while the remaining sixth are subdivided among the other five fields. Finally, for the industry of activity, Table 1 shows that one third of the respondents are active in manufacturing. Moreover, financial and insurance activities, as well as human health and social work activities, represent a large portion of the sample.

A comparison between these summary statistics and the values collected by the Swiss Federal Statistical Office (FSO) through the Survey on Professional Education^2^ suggests that our sample is not completely representative of the specific subgroup of workers having a degree from a PET college. Concretely, our sample overrepresents men and graduates in technology-related fields. Nevertheless, the average age at graduation in our sample is in line with the ones reported by of respondents is close to the one reported by the FSO.

## Estimation results

### Main results

The first three columns of Table 2 show the estimation results of the reduced-form model presented in Eq. 4. In column (4) we report the average effect of digitalization on job characteristics, while in column (5) we multiply it with the estimated coefficients. Finally, in column (6) we show the Shapley values, which describe the contribution of each regressor in the goodness-of-fit of the estimation in column (3).

**Table 2:** Estimation results

	(1)	(2)	(3)	(4)	(5)	(6)
	$$\widehat{\beta _{c}}$$	$$\widehat{\beta _{c}}$$	$$\widehat{\beta _{c}}$$	$$\widetilde{\theta _{c}}$$	$$\widehat{\beta _{c}}$$$$\widetilde{\theta _{c}}$$	Percentage of Explained Variation
C1: Dig. increases the time pressure at work	$-$ 0.0741***	$-$ 0.0683***	$-$ 0.0776***	3.23	$-$ 0.21	1.7
	(0.014)	(0.014)	(0.0139)			
C2: Dig. puts my job at risk	$-$ 0.0312**	$-$ 0.0287**	$-$ 0.0343***	1.94	$-$ 0.067	0.52
	($-$ 0.0147)	($-$ 0.015)	($-$ 0.0148)			
C3: Dig. worsens the work–life balance	$-$ 0.102***	$-$ 0.0953***	$-$ 0.102***	2.72	$-$ 0.278	3.45
	($-$ 0.0151)	($-$ 0.015)	($-$ 0.0148)			
C4: Dig. leads to a smooth transition between working hours and leisure time	$-$ 0.0056	0.00145	0.00174	3.01	0.5	0.35
	($-$ 0.0139)	($-$ 0.0139)	($-$ 0.0137)			
C5: Dig. makes my work more interesting	0.165***	0.170***	0.155***	3.4	0.527	6.51
	($-$ 0.0169)	($-$ 0.0171)	($-$ 0.0171)			
C6: Dig. reduces the proportion of repetitive tasks	0.0138	0.0182	0.0193	3.24	0.063	1.22
	($-$ 0.0137)	($-$ 0.0137)	( $-$ 0.0136)			
C7: Dig. increases my productivity	0.281***	0.271***	0.255***	3.65	0.928	11.01
	($-$ 0.0177)	($-$ 0.0177)	($-$ 0.0179)			
C8: Dig. increases my autonomy at work	0.0405**	0.0403**	0.0380**	3.15	0.119	1.94
	($-$ 0.0167)	($-$ 0.0167)	($-$ 0.0164)			
C9: Dig. enables more flexible forms of working time	0.0231**	0.0344***	0.0269**	3.25	0.087	1.48
	($-$ 0.0119)	($-$ 0.0122)	($-$ 0.0122)			
C10: Dig. simplifies interactions with colleagues and superiors	0.0595***	0.0541***	0.0548***	3.41	0.187	3.12
	($-$ 0.0148)	($-$ 0.0148)	($-$ 0.0148)			
How strongly does Dig. affect the work over the last year?			0.112***		0.386	3.24
			($$-$$ 0.0134)			
Worker characteristics		Yes	Yes			3.37
*N*	3089	3089	3089			
$R^{2}$ (%)	34.5	36.2	37.9			

Columns (1) to (3) reports the results of the OLS regression having as dependent variable the effect of digitalization on job satisfaction, which is measured on a five point Likert scale (1 = “less satisfied”, 3 = “no change”, 5 = “more satisfied”). Robust standard errors in parentheses. *$p<0.10$, **$p< 0.05$, ***$p < 0.01$. Worker characteristics is a vector of control variables as described in Table 1 plus industry dummies according to 1-digit NACE Rev. 2 classification. Column (4) shows $\widetilde{\theta _{c}}$, the average effect of digitalization on job characteristics as reported in Table 1. Column (5) multiplies the $\beta _{c}$ from column (3) with the $\widetilde{\theta _{c}}$ from column (4). Column (6) reports the Shapley values, which describe the absolute contribution of each regressor in the goodness-of-fit of the estimation in column (3)

The estimations in the first three columns differ in terms of control variables, e.g., column (1) contains no control variables. Overall, the ten characteristics explain about 34.5% of the total variance in the effect of digitalization on job satisfaction. Column (2) controls for individual characteristics, and column (3) further controls for the influence of digitalization on work in the preceding year. We find that these control variables have hardly any influence on the estimated coefficients. While the additional control variables increase the percentage of explained variance, they do so only slightly, to 37.9%.

The coefficients of the OLS regression of column (3) test the association through the channels. The value for $\widehat{\beta _{c}}$ $\widetilde{\theta _{c}}$ reported in column (5) and the Shapley values reported in column (6) allow us to quantify the importance of each channel.

We start by considering the channels of the dimension “time use.” The OLS coefficient for time pressure at work is negative and statistically significant, suggesting that the increase in time pressure at work resulting from digitalization decreases job satisfaction. Column (4) reports the corresponding value of $\widetilde{\theta _{c}}$, the impact of digitalization on time pressure, which is average. Thus, as column (5) shows, $\widehat{\beta _{c}}$ $\widetilde{\theta _{c}}$ amounts to -0.25. Column (6) shows that the increase in time pressure due to digitalization accounts for about 1.7% of the total variance. This finding implies that digitalization is associated with job satisfaction through channel C1, which suggests that an increase in time pressure at work generated by digitalization is negatively associated with job satisfaction.

The second channel of the “time use” dimension is the fear of job loss. While OLS coefficients for the fear of losing one’s job are also negative and statistically significant, they are lower than the coefficient for the increase in time pressure. Moreover, $\widetilde{\theta _{c}}$ is relatively low. Thus the resulting value of $\widehat{\beta _{c}}$ $\widetilde{\theta _{c}}$ is particularly low. This channel explains about 0.5% of the overall variance. Nevertheless, we should not interpret this result as meaning that the fear of losing one’s job has no effect on job satisfaction. Instead, in this case it means that digitalization has almost no effect on workers’ job satisfaction in terms of that fear. Thus, while we confirm the association through channel C2—that the increase in the fear of losing one’s job derived by digitalization is negatively associated with job satisfaction—we find a relatively small effect magnitude for this channel in our sample. This finding, however, should be relativized given the relatively low unemployment probability of the sample consisting of workers with a degree from a PET college.^3^

The third channel of the “time use” dimension is work-life balance. The large and negative OLS coefficient suggests that this channel has the strongest negative effect on job satisfaction of all ten channels. However, as with the previous channel, the relatively low value of $\widetilde{\theta _{c}}$ reduces the value of $\widehat{\beta _{c}}$ $\widetilde{\theta _{c}}$. Given that this channel explains about 3.4% of the total variance, we find that the relatively high value of $\widetilde{\theta _{c}}$ suggests a positive association through channel C3—that the deterioration of the work-life balance induced by digitalization is negatively associated with job satisfaction.

The final channel in the “time use” dimension is the smoothness of transition between work and private life. The OLS coefficient for this channel is not statistically different from zero, and $\theta$ remains relatively low. As the resulting $\widehat{\beta _{c}}$ $\widetilde{\theta _{c}}$ is also close to zero, this channel explains less than 0.4% of the total variance. Thus, a smoother transition between working hours and leisure due to digitalization does not affect job satisfaction. Our findings therefore do not support an association through channel C4—that the smoothing of the transition between working hours and leisure allowed by digitalization is negatively associated with job satisfaction.

For the dimension “new activities,” the OLS coefficient for the interestingness of work is positive and statistically significant. Given the high value of $\widetilde{\theta _{c}}$, the resulting $\widehat{\beta _{c}}$ $\widetilde{\theta _{c}}$ is also high. This channel explains about 6.5% of the total variance. Our estimations thus imply that digitalization is associated with job satisfaction through channel C5—that an increase in work interestingness derived from digitalization is positively associated with job satisfaction.

The second channel of the “new activities” dimension is percentage of repetitive tasks. The OLS coefficient for this channel is small and not statistically different from zero. Thus the resulting value of $\widehat{\beta _{c}}$ $\widetilde{\theta _{c}}$ is low, even though $\widetilde{\theta _{c}}$ is relatively high. This channel explains about 1.2% of the total variance. Nevertheless, the low value of $\widehat{\beta _{c}}$ suggests that the reduction in the proportion of repetitive tasks as a result of digitalization does not markedly affect job satisfaction. The association through channel C6—that reduction in the proportion of repetitive tasks induced by digitalization is positively associated with job satisfaction—is thus not confirmed. The finding that digitalization does affect repetitive tasks but that this does not translate into changes of job satisfaction, might be surprisingly in the light of the current literature (see, e.g., Castellacci and Viñas-Bardolet 2019).

The third channel of the “new activities” dimension is productivity. The large and positive OLS coefficient suggests that this channel has the strongest effect on job satisfaction. Furthermore, this channel has the largest value of $\widetilde{\theta _{c}}$, meaning that digitalization affects workers’ productivity particularly strongly. The combination of these two large values gives a very high value of $\widehat{\beta _{c}}$ $\widetilde{\theta _{c}}$, showing the large contribution of this channel to explain the effect of digitalization on job satisfaction. Indeed, this channel alone accounts for about 11% of the total variance. This result clearly supports the association through channel C7—that an increase in work interestingness derived from digitalization is positively associated with job satisfaction.

**Fig. 2:**
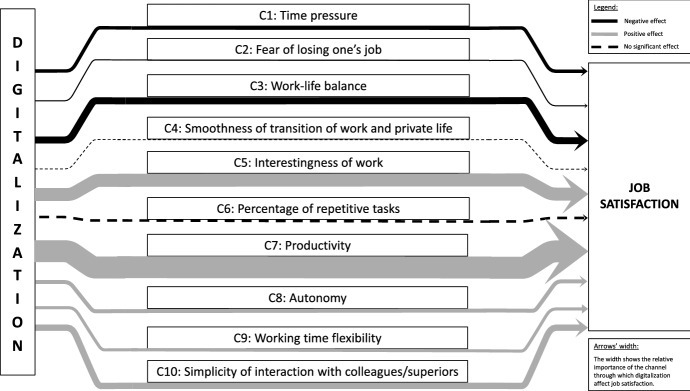
Summary of the results. *Notes*: This figure shows the relative importance of the ten channels in explaining the impact of digitalization on workers’ job satisfaction. The width of the arrows represents the relative importance of the channel. Black stands for channels with negative effects on job satisfaction. Gray represents a positive effect. A dashed arrow indicates that digitalization via this channel has no statistically significant effect on job satisfaction

The fourth channel of the “new activities” dimension is autonomy. The OLS coefficient for the increase in autonomy is positive and statistically significant. However, the relatively low value of $\widehat{\beta _{c}}$ multiplied by an average value of $\widetilde{\theta _{c}}$ gives a relatively small value of $\widehat{\beta _{c}}$ $\widetilde{\theta _{c}}$. This channel explains altogether about 1.9% of the total variance in the model, a finding suggesting that an increase in the autonomy at work derived by digitalization is positively associated with job satisfaction and thus confirms a positive association through channel C8.

As for dimension “access to information,” we observe that the OLS coefficient for the flexibility of working time is positive and statistically significant but relatively small. Given the average value of $\widetilde{\theta _{c}}$, the resulting value of $\widehat{\beta _{c}}$ $\widetilde{\theta _{c}}$ is relatively low. This indicator explains about 1.5% of the total variance. Still, this finding imply that digitalization is associated with job satisfaction through channel C9, suggesting that more flexible forms of work allowed by digitalization are positively associated with job satisfaction.

For the coefficient of the dimension “communication tools,” the OLS coefficient for the simplicity of interaction with colleagues and superiors is positive and statistically significant. Given the relatively high value of $\widetilde{\theta _{c}}$, the resulting $\widehat{\beta _{c}}$ $\widetilde{\theta _{c}}$ is also relatively high. This channel explains about 3.1% of the overall variance, meaning that more simple interaction with colleagues and superiors eased by digitalization are positively associated with job satisfaction, and thus supports the association through channel C10. Figure 2 summarizes the main findings discussed thus far.

### Heterogeneity across workers

By describing the channels in Sect. 2, we refer on the entire sample and refrain from refining them according to workers’ individual characteristics. Nevertheless, the data offers information on workers’ characteristics, which can be used to explore the heterogeneity of the results.

**Fig. 3:**
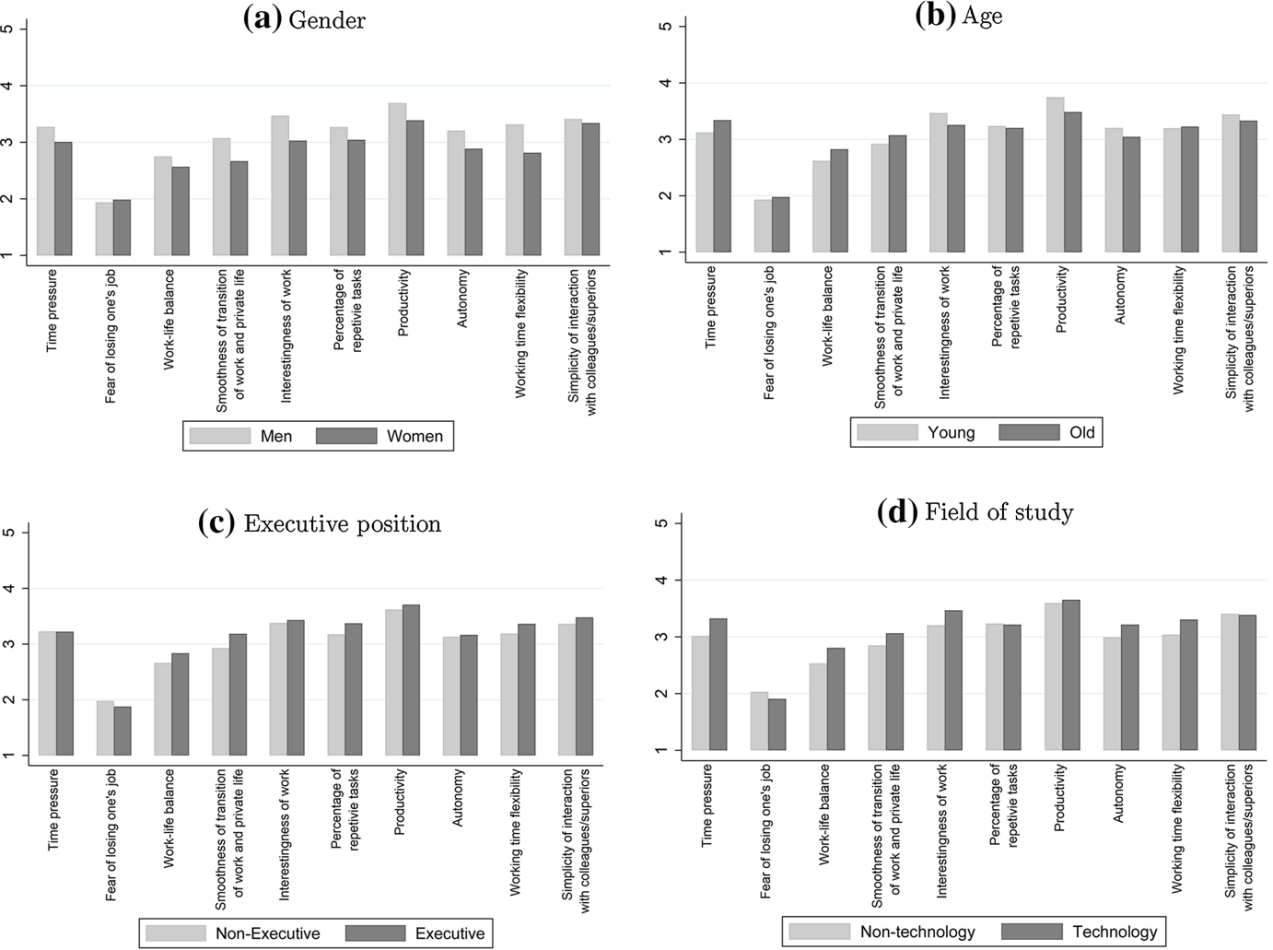
Differences in the effect of digitalization on the channels across subgroups. *Notes*: This figure shows $\theta _{c}$, the effect of digitalization on the ten channels, across subgroups of workers. These variables represent on a five-point Likert scale to what extent respondents agree with statements about the impact of digitalization on each channel. (1 = “I don’t agree at all”; 5 = “I fully agree”)

Figure 3 shows the effect of digitalization on the ten channels by subgroups of workers. In Fig. 3a we report the mean of women compared to men. This figure suggests that digitalization affects job characteristics relatively less strongly for women. The only exceptions are the effects of digitalization on increasing the fear of job loss and the simplified interaction with colleagues or superiors. These two channels are equally affected across gender. This figure suggests thus an overall weaker impact of digitalization on women compared to men.

The situation is less clear-cut when subdividing the sample by age. Figure 3b illustrates that digitalization has a relatively less strong effect on time pressure, work-life balance, and transition between work and private life for workers younger than 35 years. In contrast, digitalization increases the interestingness of work, productivity, and autonomy relatively stronger for young workers than for older ones.

Figure 3c presents the comparison of workers having a managerial position compared to workers without managerial position. Digitalization increases the fear of job loss less strongly by management workers. There is no statistical difference regarding time pressure, interestingness of work, and autonomy. In contrast, digitalization has a stronger effect on workers with managerial position with regard to the worsening of the work-life balance, the smoothing of the transition between work and private life, as well as in terms of reducing repetitive tasks and making work more flexible.

Finally, Fig. 3d compares the means of workers that graduated in a technology-related field—the one by far most diffused in our sample—compared to other workers. In this case, we observe that digitalization increases the fear of job loss relatively less strong for workers who studied in a technology-related field. In contrast, the effect of digitalization with regard to the increase in time pressure, the worsening of the work-life balance, smoothing transition between work and private life, the increase in the interestingness of work, the increase in autonomy as well as the increase in working time flexibility is relatively stronger affected compared to workers who have not studied in technology-related fields.

**Fig. 4:**
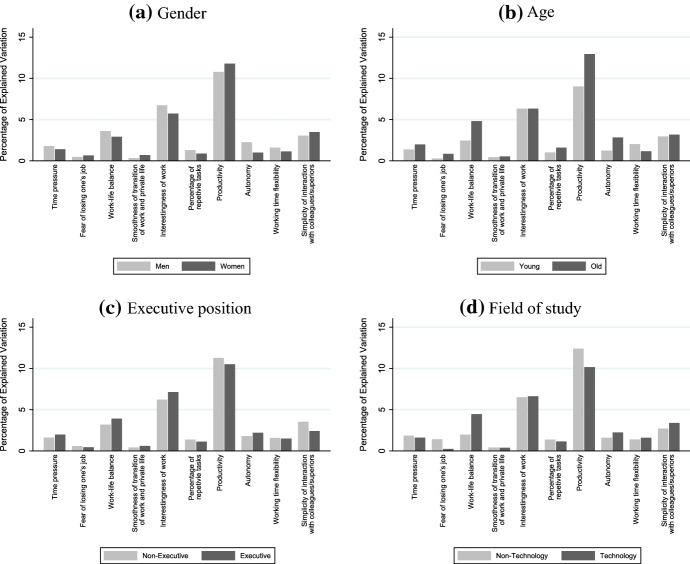
Contribution to $R^{2}$ across subsamples. *Notes*: This figure shows the percentage of variation explained by each channel across subgroups of workers. Tables 5, 6, 7 and 8 in “Appendix” reports the OLS estimates by workers’ characteristics, which underpin the regressors’ contribution to $R^{2}$

Similarly as in the previous subsection, we run the reduced-form model described in Eq. 4 for subsamples of workers according to their individual characteristics. To ease the comparisons across subgroups we report in Fig. 4 the Shapley values which allow us to quantify the relative importance of each channel. Figure 4a reports the results according to respondents’ gender; Fig. 4b according to their age; Fig. 4c according to their management position; and Fig. 4d according to their field of study. Tables 5, 6, 7 and 8 in “Appendix” reports the OLS estimates by workers’ characteristics, which underpin the regressors’ contribution to $R^{2}$.

Starting by looking at the heterogeneity across gender, Fig. 4a, shows that the channels of time pressure and particularly work-life balance are more harmful for women. In contrast, women profit more through the increase in interestingness of work and more autonomy. Finally, the productivity channel, which is the most relevant channel, is slightly less beneficial for women.

For the heterogeneity across age groups, Fig. 4b shows that the channel of losing one’s job is slightly more detrimental for older workers. Furthermore, the deterioration of the work-life balance is clearly more critical for older workers. In contrast, older workers profit more through the increase of productivity and autonomy. However, older workers benefit less from more interesting work. Similarly, the channel of working time flexibility is slightly less beneficial for older workers.

As for the heterogeneity across executive position, Fig. 4c shows that non-executive workers suffer slightly less from an increase in time pressure and the worsening of the work-life balance. Additionally, non-executive workers benefit more from the increase in productivity and from an easier interaction with colleagues and superiors. However, they profit less from more interesting work.

Finally, for the heterogeneity across field of study, Fig. 4d shows that the channel of losing one’s job is clearly more harmful for workers outside technology-related fields. However, they suffer less for the worsening of the work-life balance. Furthermore, workers outside technology-related fields profit more through the increase in productivity. Nevertheless, workers outside technology-related fields benefit less from more autonomy and easier interaction with colleagues and superiors.

### Robustness checks

To examine the robustness of our main findings, we performed several robustness tests. First, we conduct a heterogeneous treatment analysis to check whether the overall effect of digitalization on work might affect the relevance and perception of different channels. Second, we weight our baseline estimates with external information about the characteristics of PET student population. This estimation tests for possible non-representativeness of the sample, for instance, due to the fact that the data were collected through an online survey.

#### Heterogeneous treatment effects

Our main estimation controls for how strongly digitalization affected respondents’ work over the last year, thereby accounting for unobserved characteristics related to both digitalization and satisfaction. However, this information can be also used to address potential heterogeneity in the relevance and perception of different job characteristics. For example, individuals that are weakly affected by digitalization might consider the channel through time pressure particularly important, while individuals that are strongly affected by digitalization might consider the channel through fear of losing ones job particularly important. On average, the two channels appear similarly important though the effect on average job satisfaction is stronger for the second channel. The reason is that the average does not account for the correlation between the effect of digitalization on work satisfaction and the relevance of the channels.

Therefore, we conduct a robustness check that interacts the overall effect of digitalization on work with the effect of digitalization on each job characteristic. To ease interpretation, we standardize the resulting interaction terms by assigning the same mean and variance of the original effect of digitalization on each job characteristic.

**Table 3:** Robustness check: heterogeneous treatment effects

	(1)	(2)
	OLS	OLS
C1: Dig. increases the time pressure at work	$-$ 0.0776***	
	(0.0128)	
C1 * How strongly Dig. affect the work over the last year?		$-$ 0.107***
		(0.0175)
C2: Dig. puts my job at risk	$-$ 0.0343***	
	(0.0133)	
C2 * How strongly Dig. affect the work over the last year?		$-$ 0.0251
		(0.0154)
C3: Dig. worsens the work–life balance	$-$ 0.102***	
	(0.0137)	
C3 * How strongly Dig. affect the work over the last year?		$-$ 0.120***
		(0.0171)
C4: Dig. leads to a smooth transition between working hours and leisure time	0.00174	
	(0.0131)	
C4 * How strongly Dig. affect the work over the last year?		0.00758
		(0.0171)
C5: Dig. makes my work more interesting	0.155***	
	(0.0154)	
C5 * How strongly Dig. affect the work over the last year?		0.233***
		(0.0231)
C6: Dig. reduces the proportion of repetitive tasks	0.0193	
	(0.0128)	
C6 * How strongly Dig. affect the work over the last year?		0.0267
		(0.0178)
C7: Dig. increases my productivity	0.255***	
	(0.0161)	
C7 * How strongly Dig. affect the work over the last year?		0.416***
		(0.0259)
C8: Dig. increases my autonomy at work	0.0380***	
	(0.0147)	
C8 * How strongly Dig. affect the work over the last year?		0.0516**
		(0.0207)
C9: Dig. enables more flexible forms of working time	0.0269**	
	(0.0116)	
C9 * How strongly Dig. affect the work over the last year?		0.0365**
		(0.0153)
C10: Dig. simplifies interaction with colleagues and superiors	0.0548***	
	(0.0136)	
C10 * How strongly Dig. affect the work over the last year?		0.0772***
		(0.0196)
How strongly Dig. affect the work over the last year?	0.112***	$-$ 0.260***
	(0.0121)	(0.0281)
Worker characteristics	Yes	Yes
(*N*)	3089	3089
($R^{2}$)	0.379	0.381

This table reports the results of the OLS regression having as dependent variable the effect of digitalization on job satisfaction, which is measured on a five point Likert scale (1 = “less satisfied”, 3 = “no change”, 5 = “more satisfied”). Robust standard errors in parentheses. *$p < 0.10$, **$p < 0.05$, ***$p< 0.01$. Worker characteristics is a vector of control variables as described in Table 1. Column (1) reports the baseline estimation as in Table 2, while column (2) reports the estimation of the model in which we allow the channel to interact with the overall effect of digitalization on work

Table 3 shows the results of this first robustness test. Column (1) reports the baseline estimation as in Table 2, while column (2) reports the estimation of the model in which we allow the channel to interact with the overall effect of digitalization on work. The comparison of the coefficients of these two columns suggests that indeed digitalization might partially strengthen or weaken the effect of the channels. Nevertheless, the signs and the sizes of the coefficients are similar, supporting thus our baseline results.

#### Respondents’ self-selection

Non-response represents another concern. To alleviate this concern, we run a robustness test in which we weight our baseline estimates with external information about the characteristics of PET students. Concretely, the set of weights use data about the characteristics of the population of PET graduates.^4^ This allows to create estimates that are representative for the population of graduates of PET colleges.

Table 4 shows the results of this second robustness test. Column (1) reports the baseline estimation as in Table 2, while column (2) reports the estimation of the model in which we weight our baseline estimates with external information about the characteristics of PET students. The comparison of the coefficients of these two columns suggests that although the sample considered in this study is not perfectly representative, the results are close to the ones in which we correct for the lack of representativeness. The only difference is applied to the channel about the fear of job loss (C2), which in the weighted estimates presents a negative coefficient, although not anymore statistically significant. The coefficients of all other channel show values in line with the baseline estimation.

Thus, this robustness test alleviates some possible selection bias in participating in the survey based on individual characteristics such as gender and field of study.

**Table 4:** Robustness check: respondents’ self-selection

	(1)	(2)
	OLS	OLS weighted
C1: Dig. increases the time pressure at work	$-$ 0.0776***	$-$ 0.0735***
	(0.0139)	(0.0152)
C2: Dig. puts my job at risk	$-$ 0.0343**	$-$ 0.0202
	(0.0148)	(0.0161)
C3: Dig. worsens the work–life balance	$-$ 0.102***	$-$ 0.114***
	(0.0148)	(0.0163)
C4: Dig. leads to a smooth transition between working hours and leisure time	0.00174	0.00265
	(0.0137)	(0.0150)
C5: Dig. makes my work more interesting	0.155***	0.159***
	(0.0171)	(0.0189)
C6: Dig. reduces the proportion of repetitive tasks	0.0193	0.0173
	(0.0136)	(0.0145)
C7: Dig. increases my productivity	0.255***	0.243***
	(0.0179)	(0.0193)
C8: Dig. increases my autonomy at work	0.0380**	0.0424**
	(0.0164)	(0.0178)
C9: Dig. enables more flexible forms of working time	0.0269**	0.0319**
	(0.0122)	(0.0130)
C10: Dig. simplifies interaction with colleagues and superiors	0.0548***	0.0621***
	(0.0148)	(0.0156)
How strongly Dig. affect the work over the last year?	0.112***	0.113***
	(0.0134)	(0.0146)
Controls	Yes	Yes
(*N* )	3089	3089
($R^{2}$)	0.379	0.378

This table reports the results of the OLS regression having as dependent variable the effect of digitalization on job satisfaction, which is measured on a five point Likert scale (1 = “less satisfied”, 3 = “no change”, 5 = “more satisfied”). Robust standard errors in parentheses. *$p < 0.10$, **$p< 0.05$, ***$p < 0.01$. Worker characteristics is a vector of control variables as described in Table 1. Column (1) reports the baseline estimation as in Table 2, while column (2) reports a weighted estimation that accounts for the distribution of gender and field in the population of PET graduates

## Conclusion, limitations, and implications

Using graduates of PET colleges in Switzerland as a case study, this paper provides insights into the relative strength of ten channels through which digitalization affects job satisfaction. We find that digitalization increases job satisfaction among PET graduates particularly by increasing work productivity, making work more interesting, and fostering interactions with coworkers and supervisors. Relatively less important is the positive effect of digitalization on job satisfaction through the increase in workers’ autonomy and more flexible forms of work.

Our results further suggest that the worsening of the work-life balance and the increase in time pressure derived by digitalization are negatively associated with workers’ job satisfaction. Furthermore, while the widespread idea that the fear of losing one’s job to digitalization is negatively associated with job satisfaction is confirmed, it remains small in magnitude in our sample. Finally, our estimates provide no evidence that (a) the smoothing of the transition between working hours and leisure allowed by digitalization is negatively associated with job satisfaction, or (b) the reduction of repetitive tasks caused by digitalization has an effect on workers’ job satisfaction.

We further investigate the heterogeneity of our results by decomposing the sample according to respondents’ gender, age, management position, and field of study. By comparing the relative contribution of the channels, we find relatively similar patterns across subsamples. Major differences occur only for the effect that digitalization has on job satisfaction through the worsening of the work-life balance, a finding more relevant for men, for workers aged more than 35 years (roughly the average age in our sample), for workers with an executive position, and for workers whose field of study technology-related. For the effect that digitalization has on job satisfaction through an increase in the interestingness of work, we find a larger effect for males and for workers younger than 35. In contrast, the effect that digitalization has on job satisfaction through an increase in autonomy is lower for young workers, for women, and for workers who did not study in technology-related fields. In terms of productivity, we find that digitalization is more beneficial for women, for older workers, for workers without an executive position, and for workers who did not study in technology-related fields. Finally, the positive effect that digitalization has on job satisfaction by simplifying interactions with colleagues and superiors is larger for non-executive workers than for executives.

One limitation in our study is that the presented estimates are not necessarily causal. We control for gender, age, field of study, industry and management position. Furthermore, we include ten channels simultaneously and control for a measure of how strongly respondents assess the impact of digitalization on their job. The inclusion of the variable measuring the impact of digitalization on work might partially address potential omitted variable bias. Nevertheless, one could think at 10 different omitted characteristics—one for each channel—that are individually both influencing the self-reported impact of digitalization on the respondents’ job satisfaction and a respondents’ self-reported channel. However, since the self-reported impact of digitalization on the respondents’ job is unlikely to be a good proxy for all 10 different omitted characteristics, the existence of omitted variable bias cannot be completely ruled out. Therefore, future research should investigate other job characteristics that might prove to be channels for the effect of digitalization on job satisfaction.

Another limitation arises from the fact that the sample considered in this study is not perfectly representative, especially with regard to gender and field of study. Nevertheless, the fact that the results between subsamples are particularly robust partially reassures the strength of the results. Furthermore, weighting estimates with information about the population of PET graduates yield similar results. This robustness test also alleviates concerns about non-response to some extent. However, it remains possible that taking part in an online-survey might already impose a self-selection of respondents which are akin to and open to new technologies.

Additionally, our results need cautious interpretation because they are specific to the particular sample investigated in this paper, which mainly consists of workers with management-level positions. The findings may differ substantially for workers with no tertiary vocational education. For example, a recent investigation conducted by Pfrombeck et al. (2020) on a representative sample of Swiss workers shows that, on average, a high degree of digitalization in the immediate work environment has a negative effect on job satisfaction. Future research should therefore evaluate the extent to which our results hold for different types of workers (e.g., workers with no tertiary vocational education).

Finally, a last limitation lies in this paper’s reliance on respondent self-assessments of the influence of digitalization on job satisfaction and various job characteristics. The estimates are robust to our controlling for various individual characteristics and for the self-assessed impact of digitalization on work. Furthermore, the estimates provide insights into the relative strength of various channels through which digitalization affects job satisfaction. However, due to the empirical strategy of a reduced-form estimation, we are unable to interpret the results in absolute terms. Therefore, future research should use measures of digitalization, job characteristics, and job satisfaction that allow the estimating of structural models that capture these concepts directly and are less prone to potential measurement error.

The insights gained from this paper on the way in which digitalization affects job satisfaction are even more crucial in the light of the COVID-19 pandemic, which has sped up the digitalization process across the world. In particular, the massive shift to telework and the boom of digital tools use in 2020 (e.g., (DeFilippis et al. 2020) can modify the results they obtain on data collected in 2019. Further research along these lines is needed.

## Appendix

See Tables 5, 6, 7 and 8.

**Table 5:** Subsample results: estimation by gender

	Man				Women			
	(1)	(2)	(3)	(4)	(5)	(6)	(7)	(8)
	$$\widehat{\beta _{c}}$$	$$\widetilde{\theta _{c}}$$	$$\widehat{\beta _{c}}$$$$\widetilde{\theta _{c}}$$	Percentage of Explained Variation	$$\widehat{\beta _{c}}$$	$$\beta _{c}$$	$$\widehat{\beta _{c}}$$$$\widetilde{\theta _{c}}$$	Percentage of Explained Variation
C1: Dig. increases the time pressure at work	$-$ 0.0766***	3.27	$-$ 0.25	1.77	$-$ 0.0756**	3.05	$-$ 0.23	1.38
	(0.0159)				(0.0295)			
C2: Dig. puts my job at risk	$-$ 0.026	1.93	$-$ 0.05	0.46	$-$ 0.0511	2.01	$-$ 0.1	0.64
	(0.0167)				(0.0322)			
C3: Dig. worsens the work–life balance	$-$ 0.103***	2.75	$-$ 0.28	3.6	$-$ 0.100***	2.58	$-$ 0.26	2.9
	(0.0168)				(0.0314)			
C4: Dig. leads to a smooth transition between working hours and leisure time	$-$ 0.000794	3.08	0	0.31	0.00563	2.73	0.02	0.68
	(0.0155)				(0.0294)			
C5: Dig. makes my work more interesting	0.160***	3.47	0.56	6.72	0.134***	3.09	0.41	5.72
	(0.0192)				(0.039)			
C6: Dig. reduces the proportion of repetitive tasks	0.0212	3.28	0.07	1.29	0.00635	3.07	0.02	0.87
	(0.0153)				(0.0317)			
C7: Dig. increases my productivity	0.251***	3.69	0.93	10.76	0.262***	3.45	0.9	11.76
	(0.02)				(0.0395)			
C8: Dig. increases my autonomy at work	0.0468**	3.2	0.15	2.23	$-$ 0.0015	2.94	0	0.99
	(0.0186)				(0.0344)			
C9: Dig. enables more flexible forms of working time	0.0313**	3.32	0.1	1.58	0.0117	2.94	0.03	1.13
	(0.0135)				(0.0277)			
C10: Dig. simplifies interactions with colleagues and superiors	0.0546***	3.42	0.19	3.04	0.0670**	3.38	0.23	3.48
	(0.0168)				(0.0317)			
How strongly does Dig. affect the work over the last year?	0.108***			3.07	0.113***			3.59
	($-$ 0.015)				(0.0302)			
Worker characteristics	Yes			3.16	Yes			7.61
*N*	1042				2047			
$R^{2}$ (%)	37.99				40.73			

Columns (1) and (5) report the results of the OLS regression having as dependent variable the effect of digitalization on job satisfaction, which is measured on a Likert scale (1 = “less satisfied”, 3 = “no change”, 5 = “more satisfied”’). Robust standard errors in parentheses. *$p < 0.10$, **$p < 0.05$, ***$p < 0.01$. Worker characteristics is a vector of control variables as described in Table 1 plus industry dummies according to NACE 1-digit classification. Columns (2) and (6) report the subsample average values of the job characteristics. Columns (3) and (7) reports the multiplication of the betas from columns (1) and (4) with the subsample average values of the characteristics. Columns (4) and (8) report the percentage of explained variance by each regressor in the estimation in column (1)

**Table 6:** Subsample results: estimation by age

	Young ($\le$ 35 years)				Old (> 35 years)			
	(1)	(2)	(3)	(4)	(5)	(6)	(7)	(8)
	$$\widehat{\beta _{c}}$$	$$\widetilde{\theta _{c}}$$	$$\widehat{\beta _{c}}$$$$\widetilde{\theta _{c}}$$	Percentage of explained variation	$$\widehat{\beta _{c}}$$	$$\widetilde{\theta _{c}}$$	$$\widehat{\beta _{c}}$$$$\widetilde{\theta _{c}}$$	Percentage of explained variation
C1: Dig. increases the time pressure at work	$-$ 0.0768***	3.14	$-$ 0.25	1.37	$-$ 0.0790***	3.35	$-$ 0.26	1.97
	(0.0224)				(0.018)			
C2: Dig. puts my job at risk	$-$ 0.0411*	1.92	$-$ 0.05	0.28	$-$ 0.0239	1.98	$-$ 0.08	0.83
	(0.0218)				(0.0203)			
C3: Dig. worsens the work–life balance	$-$ 0.126***	2.62	$-$ 0.23	2.44	$-$ 0.0869***	2.84	$-$ 0.36	4.79
	(0.0242)				(0.0189)			
C4: Dig. leads to a smooth transition between working hours and leisure time	$-$ 0.00697	2.95	0.04	0.43	0.0125	3.09	$-$ 0.02	0.52
	(0.0209)				(0.0183)			
C5: Dig. makes my work more interesting	0.145***	3.5	0.57	6.31	0.162***	3.27	0.47	6.31
	(0.0257)				(0.0235)			
C6: Dig. reduces the proportion of repetitive tasks	0.0177	3.25	0.06	1.01	0.0181	3.22	0.06	1.58
	(0.0213)				(0.0179)			
C7: Dig. increases my productivity	0.281***	3.77	0.87	9.0	0.230***	3.48	0.98	12.93
	(0.0269)				(0.0239)			
C8: Dig. increases my autonomy at work	0.0569**	3.22	0.08	1.22	0.0243	3.06	0.17	2.83
	(0.0258)				(0.0215)			
C9: Dig. enables more flexible forms of working time	0.00806	3.24	0.14	2.0	0.0420***	3.26	0.03	1.14
	(0.0194)				(0.0156)			
C10 Dig. simplifies interactions with colleagues and superiors	0.0431**	3.47	0.22	2.95	0.0641***	3.33	0.14	3.16
	(0.0212)				(0.0206)			
How strongly does Dig. affect the work over the last year?	0.0846***			4.44	0.136***			2.37
	(0.0201)				(0.0182)			
Worker characteristics	Yes			2.11	Yes			2.55
*N*	1331				1758			
$R^{2}$ (%)	33.56				40.98			

Columns (1) and (5) report the results of the OLS regression having as dependent variable the effect of digitalization on job satisfaction, which is measured on a Likert scale (1 = “less satisfied”, 3 = “no change”, 5 = “more satisfied”). Robust standard errors in parentheses. *$p < 0.10$, **$p < 0.05$, ***$p < 0.01$. Worker characteristics is a vector of control variables as described in Table 1 plus industry dummies according to NACE 1-digit classification. Columns (2) and (6) report the subsample average values of the job characteristics. Columns (3) and (7) report the multiplication of the betas from columns (1) and (4) with the subsample average values of the characteristics. Columns (4) and (8) report the percentage of explained variance by each regressor in the estimation in column (1)

**Table 7:** Subsample results: estimation by executive position

	Non-executive				Executive			
	(1)	(2)	(3)	(4)	(5)	(6)	(7)	(8)
	$$\widehat{\beta _{c}}$$	$$\widetilde{\theta _{c}}$$	$$\widehat{\beta _{c}}$$$$\widetilde{\theta _{c}}$$	Percentage of explained variation	$$\widehat{\beta _{c}}$$	$$\widetilde{\theta _{c}}$$	$$\widehat{\beta _{c}}$$$$\widetilde{\theta _{c}}$$	Percentage of explained variation
C1: Dig. increases the time pressure at work	$-$ 0.0846***	3.24	$-$ 0.27	1.6	$-$ 0.0567**	3.21	$-$ 0.18	1.97
	(0.0164)				(0.0271)			
C2: Dig. puts my job at risk	$-$ 0.0398**	1.97	$-$ 0.08	0.57	$-$ 0.0277	1.88	$-$ 0.05	0.42
	(0.0175)				(0.028)			
C3: Dig. worsens the work– life balance	$-$ 0.101***	2.67	$-$ 0.27	3.16	$-$ 0.0956***	2.83	$-$ 0.27	3.89
	(0.0177)				(0.0285)			
C4: Dig. leads to a smooth transition between working hours and leisure time	0.0161	2.94	0.05	0.41	$-$ 0.0423	3.19	$-$ 0.13	0.59
	(0.016)				(0.0276)			
C5: Dig. makes my work more interesting	0.142***	3.39	0.48	6.2	0.192***	3.44	0.66	7.12
	(0.0198)				(0.0345)			
C6: Dig. reduces the proportion of repetitive tasks	0.0330**	3.19	0.11	1.36	$-$ 0.0106	3.36	$-$ 0.04	1.11
	(0.0158)				(0.0273)			
C7: Dig. increases my productivity	0.258***	3.63	0.94	11.26	0.253***	3.69	0.93	10.49
	(0.0206)				(0.0358)			
C8: Dig. increases my autonomy at work	0.0316	3.14	0.1	1.79	0.0431	3.16	0.14	2.18
	(0.0197)				(0.0294)			
C9: Dig. enables more flexible forms of working time	0.0206	3.2	0.07	1.56	0.0529**	3.35	0.18	1.48
	(0.0142)				(0.0249)			
C10: Dig. simplifies interactions with colleagues and superiors	0.0592***	3.38	0.2	3.52	0.0453	3.48	0.16	2.39
	(0.0175)				(0.0282)			
How strongly does Dig. affect the work over the last year?	0.123***			3.92	0.0855***			1.99
	(0.0154)				(0.028)			
Worker characteristics	Yes			3.23	Yes			5.07
*N*	2193				896			
$R^{2}$ (%)	38.58				38.71			

Columns (1) and (5) report the results of the OLS regression having as dependent variable the effect of digitalization on job satisfaction, which is measured on a Likert scale (1 = “less satisfied”, 3 = “no change”, 5 = “more satisfied”). Robust standard errors in parentheses. *$p < 0.10$, **$p < 0.05$, ***$p < 0.01$. Worker characteristics is a vector of control variables as described in Table 1 plus industry dummies according to NACE 1-digit classification. Columns (2) and (6) report the subsample average values of the job characteristics. Columns (3) and (7) reports the multiplication of the betas from columns (1) and (4) with the subsample average values of the characteristics. Columns (4) and (8) report the percentage of explained variance by each regressor in the estimation in column (1)

**Table 8:** Subsample results: estimation by field of study

	Non-technology				Technology			
	(1)	(2)	(3)	(4)	(5)	(6)	(7)	(8)
	$$\widehat{\beta _{c}}$$	$$\widetilde{\theta _{c}}$$	$$\widehat{\beta _{c}}$$$$\widetilde{\theta _{c}}$$	Percentage of explained variation	$$\widehat{\beta _{c}}$$	$$\beta _{c}$$	$$\widehat{\beta _{c}}$$$$\widetilde{\theta _{c}}$$	Percentage of explained variation
C1: Dig. increases the time pressure at work	$-$ 0.0904***	3.05	$-$ 0.28	1.83	$-$ 0.0685***	3.32	$-$ 0.23	1.59
	(0.0231)				(0.0172)			
C2 Dig. puts my job at risk	$-$ 0.0772***	2.04	$-$ 0.16	1.4	$-$ 0.00704	1.89	$-$ 0.01	0.22
	(0.0249)				(0.0181)			
C3: Dig. worsens the work– life balance	$-$ 0.0615**	2.55	$-$ 0.16	1.96	$-$ 0.127***	2.8	$-$ 0.36	4.44
	(0.0252)				(0.0183)			
C4: Dig. leads to a smooth transition between working hours and leisure time	$-$ 0.00343	2.9	$-$ 0.01	0.41	0.00411	3.07	0.01	0.36
	(0.0243)				(0.0168)			
C5: Dig. makes my work more interesting	0.151***	3.25	0.49	6.49	0.154***	3.48	0.54	6.61
	(0.0305)				(0.0209)			
C6: Dig. reduces the proportion of repetitive tasks	0.0321	3.26	0.1	1.36	0.0147	3.23	0.05	1.14
	(0.0257)				(0.0161)			
C7: Dig. increases my productivity	0.278***	3.63	1.01	12.38	0.237***	3.65	0.87	10.14
	(0.0313)				(0.0214)			
C8: Dig. increases my autonomy at work	0.0237	3.02	0.07	1.57	0.0476**	3.21	0.15	2.23
	(0.0285)				(0.0199)			
C9: Dig. enables more flexible forms of working time	0.0113	3.13	0.04	1.38	0.0321**	3.31	0.11	1.58
	(0.0214)				(0.0145)			
C10: Dig. simplifies interactions with colleagues and superiors	0.0327	3.44	0.11	2.68	0.0687***	3.4	0.23	3.38
	(0.0265)				(0.0174)			
How strongly does Dig. affect the work over the last year?	0.110***			3.2	0.113***			3.25
	(0.0237)				$-$ 0.016			
Worker characteristics	Yes			0.0442	Yes			3.3
*N*	1042				2047			
$R^{2}$ (%)	39.06				38.25			

Columns (1) and (5) report the results of the OLS regression having as dependent variable the effect of digitalization on job satisfaction, which is measured on a Likert scale (1 = “less satisfied”, 3 = “no change”, 5 = “more satisfied”). Robust standard errors in parentheses. *$p<0.10$, **$p<0.05$, ***$p<0.01$. Worker characteristics is a vector of control variables as described in Table 1 plus industry dummies according to NACE 1-digit classification. Columns (2) and (6) report the subsample average values of the job characteristics. Columns (3) and (7) reports the multiplication of the betas from columns (1) and (4) with the subsample average values of the characteristics. Columns (4) and (8) report the percentage of explained variance by each regressor in the estimation in column (1)

## Footnotes

1 Estimations based solely on graduates provides qualitatively similar results as those on the whole sample.

2 https://www.bfs.admin.ch/bfs/en/home/statistics/education-science/diploma/tertiary-advanced-professional-training.html.

3 See the unemployment rate of workers with tertiary professional education  https://www.bfs.admin.ch/bfs/de/home/statistiken/bildung-wissenschaft/bildungsindikatoren/themen/wirkung/arbeitsmarktstatus.assetdetail.12527130.html.

4 We retrieve the data on the number of PET graduates by gender and field of study from the Statistics on pupils and students (SDL) conducted by the FSO in the academic year 2019/2020.
